# Development of an activatable far-red fluorescent probe for rapid visualization of hypochlorous acid in live cells and mice with neuroinflammation

**DOI:** 10.3389/fchem.2024.1355238

**Published:** 2024-02-02

**Authors:** Long Mi, Changhe Niu, Jianqiang Chen, Feng Han, Xueying Ji

**Affiliations:** ^1^ Department of Radiology, Department of Ophthalmology, The First Affiliated Hospital of Hainan Medical University, Hainan Medical University, Haikou, China; ^2^ Wuhan Children’s Hospital, Tongji Medical College, Huazhong University of Science and Technology, Wuhan, China

**Keywords:** far-red fluorescent probe, hypochlorous acid, oxidative stress, LPS-induced neuroinflammation, bioimaging

## Abstract

Recent investigations have suggested that abnormally elevated levels of HOCl may be tightly related to the severity of neuroinflammation. Although some successes have been achieved, fluorescent probes with far-red fluorescence emission and capable of detecting HOCl with high specificity in pure aqueous solution are still urgently needed. Herein, a responsive far-red fluorescent probe, DCI-H, has been constructed to monitor HOCl activity *in vivo* and *in vitro*. DCI-H could rapidly respond to HOCl within 120 s and had a low detection limit for HOCl of 1.5 nM. Importantly, physiologically common interfering species, except for HOCl, did not cause a change in the fluorescence intensity of DCI-HOCl at 655 nm. The results of confocal imaging demonstrated the ability of DCI-H to visualize endogenous HOCl produced by MPO-catalyzed H_2_O_2_/Cl^−^ and LPS stimulation. With the assistance of DCI-H, upregulation of HOCl levels was observed in the mice model of LPS-induced neuroinflammation. Thus, we believed that DCI-H provided a valuable tool for HOCl detection and diagnosis of inflammation-related diseases.

## Introduction

Reactive oxygen species (ROS) are a class of anions, free radicals, and neutral small molecules generated by the normal metabolism of intracellular oxygen, including superoxide anion, peroxynitrite, singlet oxygen, hydrogen peroxide, hypochlorous acid (HOCl) and so on (Sies and Jones, 2020; Sun et al., 2020). Endogenous ROS can act as second messengers and are involved in regulating a series of physiological processes, such as redox balance and signal transduction (Linley et al., 2012; Ye et al., 2015; Sies et al., 2022). In living organisms, HOCl is an important ROS, mainly produced in the respiratory burst process of neutrophils (Elbim and Lizard, 2009; Güngör et al., 2009). A large body of research evidence shows that low concentrations of HOCl in organisms can regulate redox balance and resist the invasion of pathogens, while high concentrations of HOCl can cause oxidative damage to biologically active molecules (Goud et al., 2008; Guo et al., 2020; Shakya et al., 2023). For example, overexpression of HOCl can not only react with amnio, sulfhydryl, and thioethers groups to destroy the spatial structures of proteins and lead to protein inactivation but also react with nitrogen-containing bases to make nucleic acid lose the ability to assemble biological macromolecules (Dharmaraja, 2017). Hydrogen peroxide (H_2_O_2_) and chloride ion react to generate endogenous HOCl under the catalysis of myeloperoxidase (MPO) (Winterbourn and Kettle, 2000; Ulfig and Leichert, 2021), and the change of HOCl level is closely associated with the occurrence and development of numerous diseases, such as arthritis (Stamp et al., 2012), liver cirrhosis (Whiteman et al., 2005; Jaeschke and Hasegawa, 2006), kidney injury (Malle et al., 2003), and neuronal degeneration (Yap et al., 2006; Ray and Katyal, 2016). Neuroinflammation is associated with chronically activated glial cells (astrocytes and microglia) in the brain, a process that generates large amounts of ROS (Wang et al., 2018; Shukuri et al., 2021). Therefore, the development of a rapid and sensitive method for detecting HOCl is of great significance for diagnosing neuroinflammation.

Currently, routine detection of HOCl includes high-performance liquid chromatography, electrochemistry, gas chromatography, and capillary electrophoresis. However, these techniques are unable to detect HOCl directly and *in situ* in cells or *in vivo* (Zhang et al., 2018). To circumvent this limitation, small-molecule fluorescent probes have attracted much attention due to their high sensitivity, rapid response, simplicity, and excellent selectivity. Numerous HOCl fluorescent probes based on diverse fluorophores, such as coumarin (Samanta and Govindaraju, 2019; He et al., 2020a; He et al., 2020b; Ma et al., 2020; Nguyen et al., 2020; Nie et al., 2020), naphthalimide (Cheng et al., 2021; Fang and Dehaen, 2021; Wang et al., 2021; Tian et al., 2022; Wang et al., 2022), rhodamine (Lu et al., 2020; Yang et al., 2020; Zeng et al., 2023), and boron dipyrromethene (Shi et al., 2021; Bi et al., 2023; Mahanta et al., 2023), have been developed successively. The widespread use of some of the reported fluorescent probes in biological applications was still constrained by certain factors, for example, the limitation of fluorescence emission wavelengths to the visible region or the need for large amounts of organic solvents in the detection system (Dong et al., 2020; Kwon et al., 2022).

With those in mind, a novel far-red fluorescent probe DCI-H was developed. This probe exhibited excellent selectivity, fast response, and high sensitivity to HOCl. In PBS solution, HOCl promoted the release of fluorophore DCI-OH from DCI-H through a specific removal reaction of N, N-dimethylthiocarbamate, resulting in fluorescence enhancement at 655 nm. Noticeably, DCI-H had low cytotoxicity and successfully imaged endogenous HOCl in cells. In addition, the far-red fluorescence emission of DCI-H achieved real-time imaging of HOCl in mice models of LPS-induced neuroinflammation using DCI-HOCl.

## Results and discussion

### Design and synthesis of DCI-H

**SCHEME 1:**
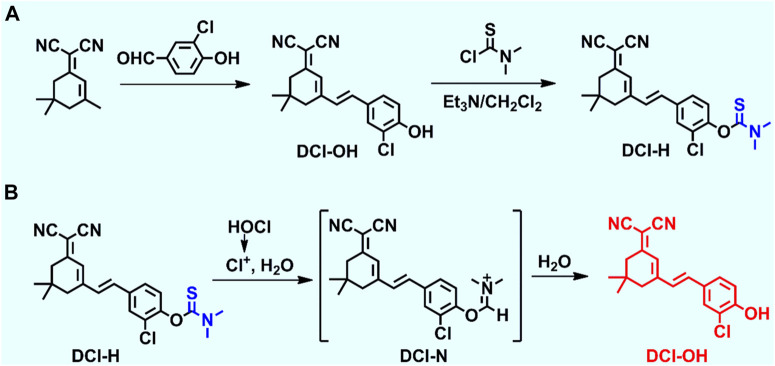
**(A)** Synthetic route of DCI-H. **(B)** The proposed reaction mechanism of DCI-H toward HOCl.

Dicyanoisophorone (DCI) was a common fluorophore used for the construction of fluorescent probes with large Stoke shifts, far-red emission properties, superior photostability, and convenient synthesis methods. A novel responsive fluorescent probe, DCI-H, was designed using DCI as the core fluorophore and N, N-dimethylthiocarbamate as the HOCl recognition site (Scheme 1A). With the introduction of chlorine atoms into the molecular backbone of DCI-OH, the pKa value could be lowered to ensure that its fluorescence emission under physiological pH conditions remained in the far-red region (Wang et al., 2020). The chemical structure of DCI-H was well characterized by 1H NMR, 13C NMR, and MS (Supplementary Figure S1–S4). The sensing mechanism of DCI-H toward HOCl was shown in Scheme 1B. Specifically, HOCl first oxidized N, N-dimethylthiocarbamate to form intermediate DCI-N, followed by the release of DCI-OH via a hydrolysis reaction. In order to further confirm this sensing process, mass spectrometry was applied to analyze the solution of DCI-H in the absence or presence of HOCl. Supplementary Figure S4 clearly showed that DCI-H itself exhibited a major peak at m/z = 410.1010, corresponding to DCI-H ([M-H]-); the product after reaction with HOCl demonstrated a major peak at m/z = 323.0890, corresponding to DCI-OH ([M-H]-) (Supplementary Figure S5).

### Spectral response of DCI-H toward HOCl

**FIGURE 1:**
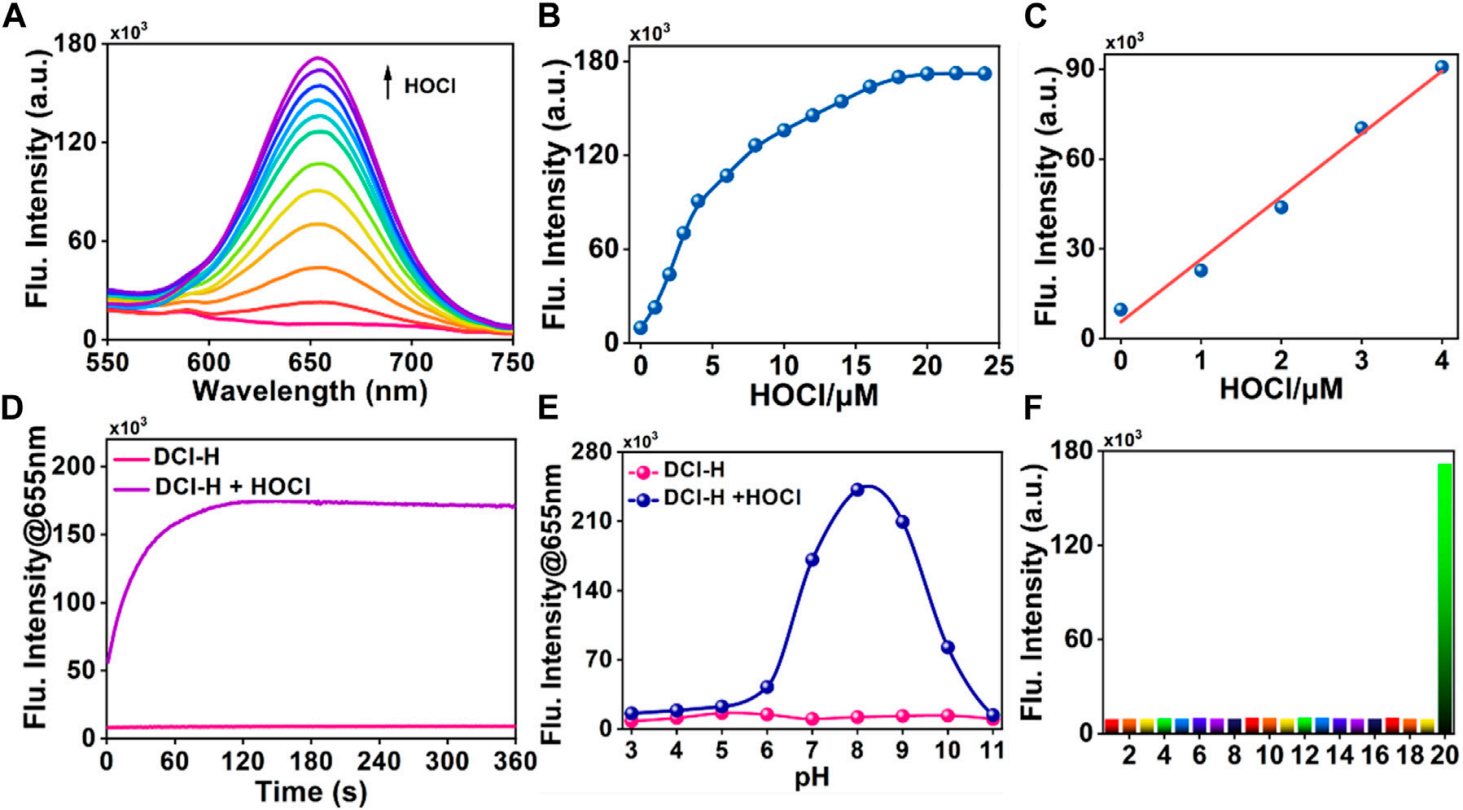
Fluorescence response of DCI-H toward HOCl. **(A)** Fluorescence spectra of DCI-H (10 μM) upon exposure to various HOCl concentrations (0, 1, 2, 3, 4, 6, 8, 10, 12, 14, 16, 18 μM). **(B)** Fluorescence intensities at 655 nm as a function of HOCl concentrations (0–24 μM). **(C)** Linearity between fluorescence intensity of DCI-H at 655 nm and HOCl concentration (0–4 μM). **(D)** Time-dependent fluorescence change of DCI-H at 655 nm in the presence or absence of HOCl (18 μM). **(E)** Effects of pH on the fluorescence of DCI-H at 655 nm in the presence or absence of HOCl (18 μM). **(F)** Fluorescence intensities of DCI-H at 655 nm in the presence of a series of potential interfering analytes (100 μM): 1) Met, 2) Gly, 3) Glu, 4) His, 5) S^2-^, 6) S_2_O_3_^2-^, 7) SO_3_^2-^, 8) SO_4_^2-^, 9) F^−^, 10) K^+^, 11) Na^+^, 12) Mg^2+^, 13) Al^3+^, 14) Ca^2+^, 15) Fe^3+^, 16) H_2_O_2_, 17) ^1^O_2_, 18) Cys, 19) GSH, 20) HOCl (18 μM). λ_ex_ = 490 nm.

Having obtained DCI-H, its spectral properties were investigated in PBS solution (pH 7.4, 10 mM, 1% acetonitrile) in detail. With 490 nm as the excitation wavelength, the blank DCI-H exhibited weak fluorescence (Figure 1A). This property provided a low background starting state for subsequent experiments to observe the HOCl-induced fluorescence changes. The fluorescence intensity of DCI-H was gradually boosted upon increasing the HOCl concentration from 0 μM to 18 μM (Figure 1B). The addition of 18 μM HOCl induced a fluorescence enhancement at 655 nm up to 17-fold compared with DCI-H itself. Such a significant fold change in fluorescence intensity indicated that DCI-H was highly sensitive for *in vitro* detection of HOCl and held the potential to be utilized in cellular and *in vivo* experiments. The fluorescence intensity value of DCI-H at 655 nm was selected to establish the relationship with HOCl concentration. The fluorescence intensity varied significantly in the presence of 1 μM HOCl while saturating at 18 μM. Data analysis further confirmed that there was a linear relationship between fluorescence intensity at 655 nm and HOCl concentration (0–4 μM), with a fitting equation of F_665_ = 20,939.7 [HOCl] + 5594.21 (R^2^ = 0.99). The limit of detection was found to be 1.5 nM in terms of the equation of LOD = 3δ/k (Figure 1C). The fluorescence intensity of DCI-H at 655 nm under continuous excitation with 490 nm light changed little within 360 s, which proved that the N, N-dimethylthiocarbamate did not undergo hydrolysis and displayed excellent stability. Upon the addition of HOCl, the fluorescence intensity increased rapidly within 60 s and remained stable after 120 s, suggesting that DCI-H had an extremely fast response to HOCl (Figure 1D). DIC-H showed negligible changes in fluorescence intensity over a wide pH range from 3.0 to 11.0, indicating that it is not susceptible to pH interference from the biological environment. When HOCl was added to the solution of DIC-H in different pH values, its fluorescence response at pH 3.0–6.0 was weak; however, the response was pronounced in the pH range of 6.0–11.0, especially reaching a maximum at pH 8.0–9.0 (Figure 1E). At physiological pH 7.4, the fluorescence response of DIC-H to HOCl was sufficient for subsequent biological experiments. Selectivity was a very important factor in evaluating the performance of DCI-H. As shown in Figure 1F, DCI-H gave weak fluorescence responses to common biological species, such as amino acids (Met, Gly, Glu, His), anions (S^2-^, S_2_O_3_^2-^, SO_3_^2-^, SO_4_^2-^, F^−^), metal ions (K^+^, Na^+^, Mg^2+^, Al^3+^, Ca^2+^, Fe^3+^), reactive oxygen species (H_2_O_2_, ^1^O_2_), and thiols (Cys, GSH). Only HOCl triggered an intensive fluorescence response, which ensured its potential for application in complex biological samples.

### *In situ* monitoring endogenous HOCl activity in live cells

**FIGURE 2:**
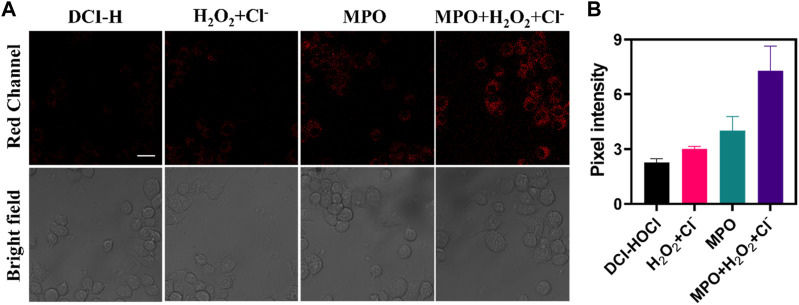
Confocal fluorescence images of RAW264.7 cells. **(A)** The cells were incubated with DCI-H (10 μM) for 30 min or preincubated with 100 μM H_2_O_2_/1 mM Cl-, 100 ng/mL MPO, 100 μM H_2_O_2_/1 mM Cl-/100 ng/mL MPO for 1 h respectively, followed by incubation with DCI-H for 30 min. **(B)** Pixel intensity from images of **(A)**. Data are presented as mean values with ±s.d. (n = 3). Scale bar: 20 μm.

Considering the high selectivity and sensitivity of DCI-H, its potential application for direct intracellular monitoring of HOCl was further evaluated. First of all, to confirm biocompatibility, RAW264.7 cells were treated with varying concentrations of DCI-H and cell viability was examined. As depicted in Supplementary Figure S6, after 24 h of incubation, the cells did not show significant cytotoxicity even at a concentration of 30 μM. MPO catalyzed the reaction between H_2_O_2_ and chloride ions to produce HOCl, which had a potent oxidizing capacity. This excess of MPO and HOCl might cause oxidative stress and damage to cells. In Figure 2, DCI-H fluoresced relatively weakly in the red channel after incubation with cells alone for 30 min. In contrast, the red channel fluorescence was slightly enhanced upon incubation with H_2_O_2_/Cl- or MPO. After treatment with MPO, H_2_O_2_, and Cl-, the red channel fluorescence was significantly augmented, suggesting that MPO catalyzed the generation of a large amount of endogenous HOCl by H_2_O_2_/Cl-. These data demonstrated that DCI-H could effectively image MPO-generated HOCl in living cells and was expected to serve as a simple tool to explore the different states of the cellular microenvironment with respect to the endogenous HOCl level.

**FIGURE 3:**
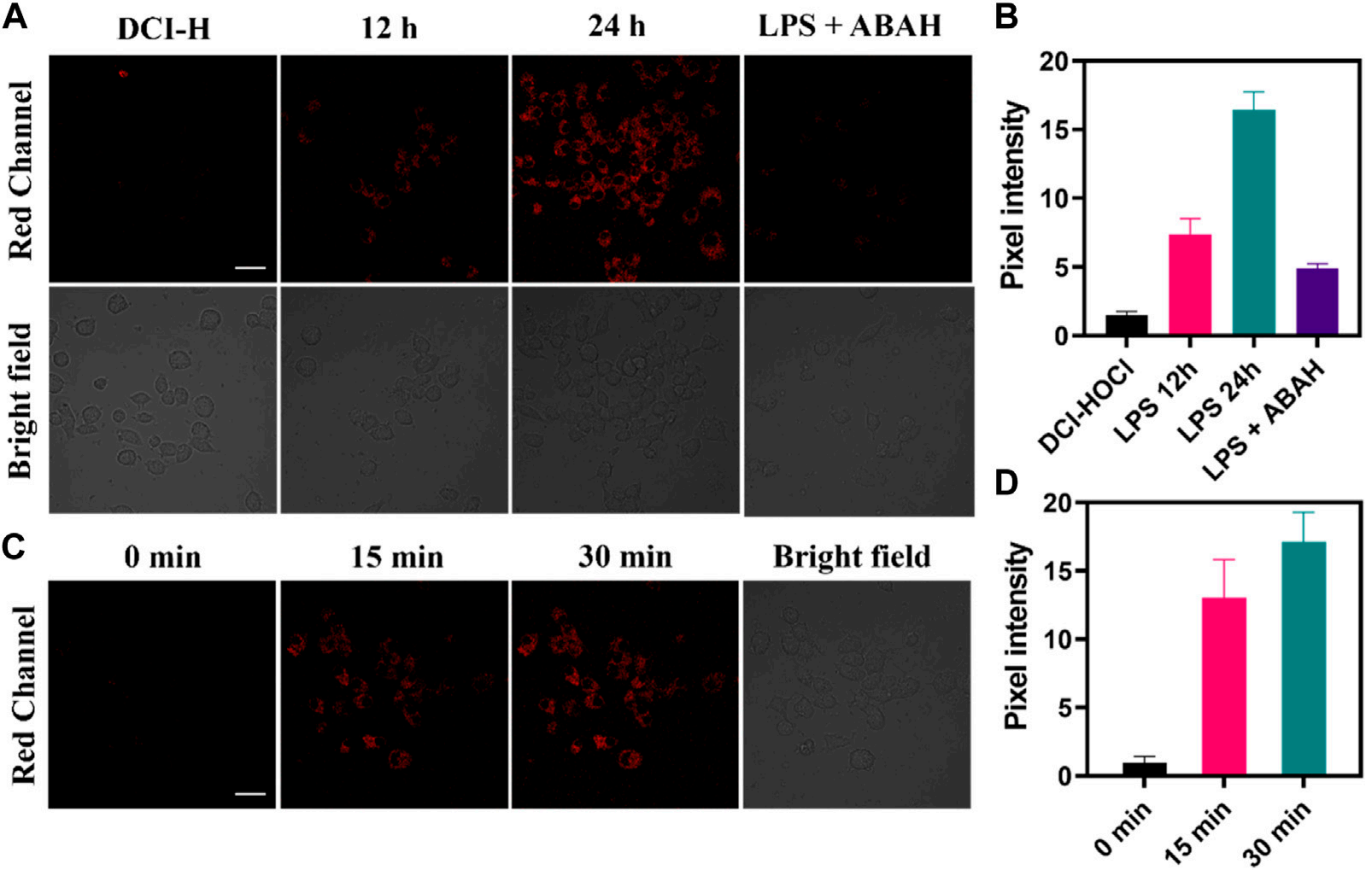
Confocal fluorescence images of RAW264.7 cells. **(A)** The cells were stimulated with 0.5 μg mL−1 LPS for 12 h, 24 h or 0.5 μg mL−1 LPS/100 μM ABAH for 24 h, and then incubated with DCI-H (10 μM) for 30 min. **(B)** Pixel intensity from images of **(A)**. **(C)** The cells were stimulated with 0.5 μg mL−1 LPS for 24 h, and then incubated with DCI-HOCl for 0 min, 15 min, 30 min. **(D)** Pixel intensity from images of **(C)**. Data are presented as mean values with ±s.d. (n = 3). Scale bar: 20 μm.

The oxidative stress process was accompanied by the upregulation of HOCl levels, so the application of DCI-H in the LPS-induced cellular inflammation model was explored. As shown in Figure 3A, incubating RAW264.7 cells with DCI-H for 30 min, only very weak fluorescence was observed in the red channel. The cells were stimulated with LPS for 12 h or 24 h followed by incubation with DCI-H for 30 min, and the red channel fluorescence was apparently strengthened compared with that of the DCI-H group. The intensity of fluorescence in the red channel of LPS-treated cells for 24 h was higher than that for 12 h, which indicated that the longer the time of LPS-stimulated cells, the higher the level of endogenous HOCl (Figure 3B). 4-aminobenzhydrazide, an inhibitor of MPO, was able to inhibit the production of HOCl inside the cell. Upon treatment of cells with LPS/ABAH for 24 h, fluorescence in the red channel was suppressed. Subsequently, real-time dynamic imaging of HOCl by DCI-H was investigated at the cellular level. The cells were first stimulated with LPS for 24 h to induce upregulation of HOCl levels followed by incubating with DCI-H and imaged at 0 min, 15 min, and 30 min (Figure 3C). It can be seen that DCI-H showed a gradual enhancement trend with the extension of time (Figure 3D). The above experimental results of confocal fluorescence imaging indicated that DCI-H could be indicative of the detection of HOCl levels in LPS-induced cell models.

### Fluorescence imaging of LPS-induced neuroinflammation *in vivo*

**FIGURE 4:**
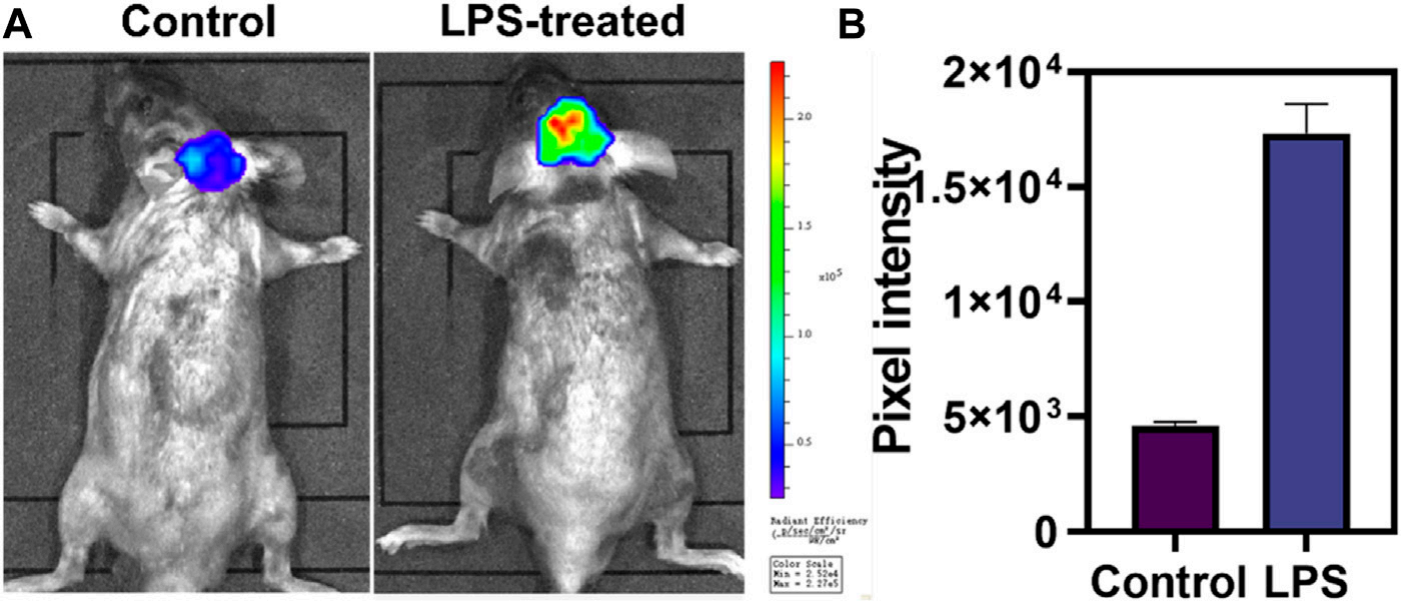
Evaluation of HOCl in neuroinflammation with DCI-H. **(A)** The normal and LPS-induced neuroinflammation mice were intracranially injected with DCI-H, respectively. **(B)** Pixel intensity from images of **(A)**. Data are presented as mean values with ±s.d. (n = 3).

Motivated by the superior performance in cellular imaging, we intended to validate the ability of DCI-H to monitor HOCl in a model of neuroinflammation. LPS triggered a series of microglial cell responses to cause neuroinflammation by interacting with the membrane receptor Toll-like receptor 4 (Allendorf et al., 2020). In light of this, C57BL/6J mice were injected intraperitoneally with LPS to mimic neuroinflammation. As illustrated in Figure 4, normal mice were injected intracranially with DCI-H and imaged 30 min later, which revealed weak fluorescence in the mice’s brains. Surprisingly, when mice were injected intraperitoneally with LPS for a week to induce neuroinflammation, followed by intracranial injection of DCI-H, significant fluorescence enhancement was observed in the brains of mice. The above experimental results demonstrated that HOCl expression level was upregulated in the brains of mice with LPS-induced neuroinflammation, and DCI-H was able to image this process dynamically.

## Conclusion

In summary, a responsive far-red fluorescent probe, DCI-H, was designed and synthesized, which could be directly employed for the detection of HOCl activity. *In vitro* analysis in PBS solution revealed that DCI-H had ultra-high sensitivity, rapid response, and high selectivity for HOCl. Most importantly, it could also dynamically monitor the fluctuation of intracellular HOCl levels in real time via DCI-H. DCI-H was successfully applied to *in vivo* imaging of LPS-induced neuroinflammation model mice, from which it was found that the levels of HOCl in the brains of neuroinflammatory mice were significantly elevated and much higher than that of normal mice. Thus, DCI-H had the potential to be exploited as an effective chemical tool for discovering the biological functions of HOCl and related disease diagnosis.

### Materials and general experimental methods

For details, see Supplementary Material.

Synthesis of (E)-O-(2-chloro-4-(2-(3-(dicyanomethylene)-5,5-dimethylcyclohex-1 -en-1-yl)vinyl) phenyl) dimethylcarbamothioate (DCI-H). DCI-OH was synthesized according to the previously reported procedure. DCI-OH (324 mg, 1.0 mmol) was dissolved in dry dichloromethane (10 mL), and then the reaction system was cooled to 0 °C. Diethylamino thionyl chloride (369 mg, 3.0 mmol) and triethylamine (0.5 mL) were added slowly and sequentially. The reaction mixture was brought to room temperature and stirred overnight. The reaction was monitored by TLC. Upon completion of the reaction, the solvent was removed by distillation under reduced pressure. The resulting product was purified by silica gel column chromatography (eluent gradient: 25% ethyl acetate/75% petroleum ether) to give the desired compound (205 mg, 50% yield). ^1^H NMR (400 MHz, CDCl_3_): δ 7.56 (s, 1H), 7.42 (d, *J* = 8.4 Hz, 1H), 7.17 (d, *J* = 8.4 Hz, 1H), 6.94 (s, 2H), 6.84 (s, 1H), 3.46 (s, 3H), 3.38 (s, 3H), 2.59 (s, 2H), 2.43 (s, 2H), 1.07 (s, 6H); ^13^C NMR (100 MHz, CDCl_3_): δ 185.96, 169.04, 153.01, 150.49, 134.83, 134.50, 130.36, 128.91, 128.41, 126.33, 125.70, 124.23, 113.29, 112.49, 79.43, 43.48, 42.92, 39.12, 38.92, 32.01, 27.97; HRMS m/z: C_22_H_22_ClN_3_OS [M-H]^+^ calcd for 410.1094 found 410.1010.

## Data Availability

The original contributions presented in the study are included in the article/Supplementary Material, further inquiries can be directed to the corresponding author.
