# Supplementary material for: Development of an activatable far-red fluorescent probe for rapid visualization of hypochlorous acid in live cells and mice with neuroinflammation
Source: Front Chem. 2024 Feb 2;12:1355238. doi: 10.3389/fchem.2024.1355238 (PMC10869478; doi:10.3389/fchem.2024.1355238)
Supplement: Supplementary file 1 [file DataSheet1.docx]

**Supporting Information**

**Development of an activatable far-red fluorescent probe for rapid visualization of hypochlorous acid in live cells and mice with neuroinflammation**

Long Mi^1, #^, Changhe Niu^2, #^, Jianqiang Chen^1^, Feng Han^1^, Xueying Ji^1, *^

^1^ Department of Radiology, Department of Ophthalmology, The First Affiliated Hospital of Hainan Medical University, Hainan Medical University, Haikou, 570102, China

^2^ Wuhan Children's Hospital, Tongji Medical College, Huazhong University of Science and Technology, Wuhan 430016, China

^*^To whom correspondence should be addressed.

E-mail: [jixueying@163.com](mailto:jixueying@163.com) (X. Y. J.)

^#^These two authors contributed equally to this work (L. Mi and C. H. Niu).

**Contents:**

1. **General information**
2. **Cell culture and fluorescence imaging**
3. **Fluorescence imaging of HOCl in mice of LPS-induced neuroinflammation**
4. **Structure characterization of DCI-H**
5. **The cytotoxicity of DCI-H in living cells**

**1. General information**

Unless otherwise noted, all reagents were purchased from commercial suppliers that were used without further purification. NMR spectra were performed on a Bruker unit (400 MHz) using CDCl_3_ as the solvent. Mass spectra were obtained on Waters Xevo G2-XS Qtof mass spectrometer. Fluorescence spectra were measured on a Horiba spectrofluorometer. Cell images were performed on an Olympus FV3000 confocal laser scanning microscopy with a 60 × oil objective lens. *In vivo* images were acquired on an IVIS Lumina XR small animal optical imaging system.

**2. Cell culture and fluorescence imaging**

RAW264.7 cells were purchased from the Cell Bank of the Chinese Academy of Sciences (Shanghai, China). For cell imaging experiment, RAW264.7 cells were cultured in DMEM (Dulbecco's Modified Eagle Medium) supplemented with 10% fetal bovine serum (FBS) and 1% antibiotics. All cells were cultured in a humidified environment containing 5% CO_2_ at 37°C. Cell images were obtained on an Olympus FV3000 laser confocal microscope with the excitation at 488 nm with an objective lens (× 60). λ_ex_ = 488 nm, λ_em_ = 600 - 700 nm.

**3. Fluorescence imaging of HOCl in mice of LPS-induced neuroinflammation**

C57BL/6J mice were purchased from Nanjing University. The procedures were approved by the Institutional Animal Care and Use Committee of Hainan Medical University (protocol code HYLL-2021-133 and date of approval April 12, 2021). C57BL/6J mice were randomly divided into three groups. The control group was injected intraperitoneally with 5 mg/kg saline per day for 7 consecutive days, and the experimental group was injected intraperitoneally with 0.25 mg/kg LPS (saline soluble) per day for 7 days to induce neuroinflammation. After anesthesia, all mice were intracranially injected with DCI-H, and the brain was imaged with a small animal imaging system. Small animal imaging test conditions: λ_ex_ = 520 nm, λ_em_ = 650 nm.

**4. Structure characterization of DCI-H**





**Figure S1.** The general synthetic routes for DCI-H.

DCI-OH was synthesized according to the previously reported procedure. DCI-OH (324 mg, 1.0 mmol) was dissolved in dry dichloromethane (10 ml), and then the reaction system was cooled to 0 °C. Diethylamino thionyl chloride (369 mg, 3.0 mmol) and triethylamine (0.5 ml) were added slowly and sequentially. The reaction mixture was brought to room temperature and stirred overnight. The reaction was monitored by TLC. Upon completion of the reaction, the solvent was removed by distillation under reduced pressure. The resulting product was purified by silica gel column chromatography (eluent gradient: 25 % ethyl acetate/75 % petroleum ether) to give the desired compound (205 mg, 50 % yield). ^1^H NMR (400 MHz, CDCl_3_): δ 7.56 (s, 1H), 7.42 (d, *J* = 8.4 Hz, 1H), 7.17 (d, *J* = 8.4 Hz, 1H), 6.94 (s, 2H), 6.84 (s, 1H), 3.46 (s, 3H), 3.38 (s, 3H), 2.59 (s, 2H), 2.43 (s, 2H), 1.07 (s, 6H); ^13^C NMR (100 MHz, CDCl_3_): δ 185.96, 169.04, 153.01, 150.49, 134.83, 134.50, 130.36, 128.91, 128.41, 126.33, 125.70, 124.23, 113.29, 112.49, 79.43, 43.48, 42.92, 39.12, 38.92, 32.01, 27.97; HRMS m/z: C_22_H_22_ClN_3_OS [M-H]^+^ calcd for 410.1094 found 410.1010.


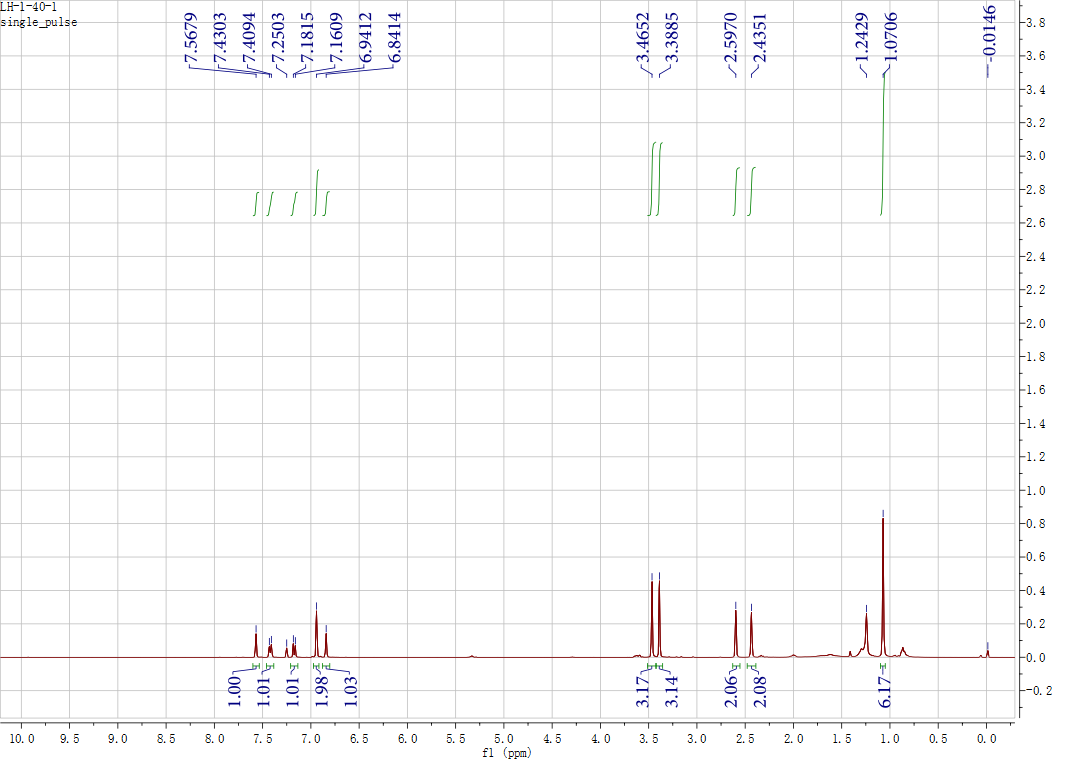


**Figure S2**. ^1^H NMR (400 MHz) spectra of DCI-H in CDCl_3_.


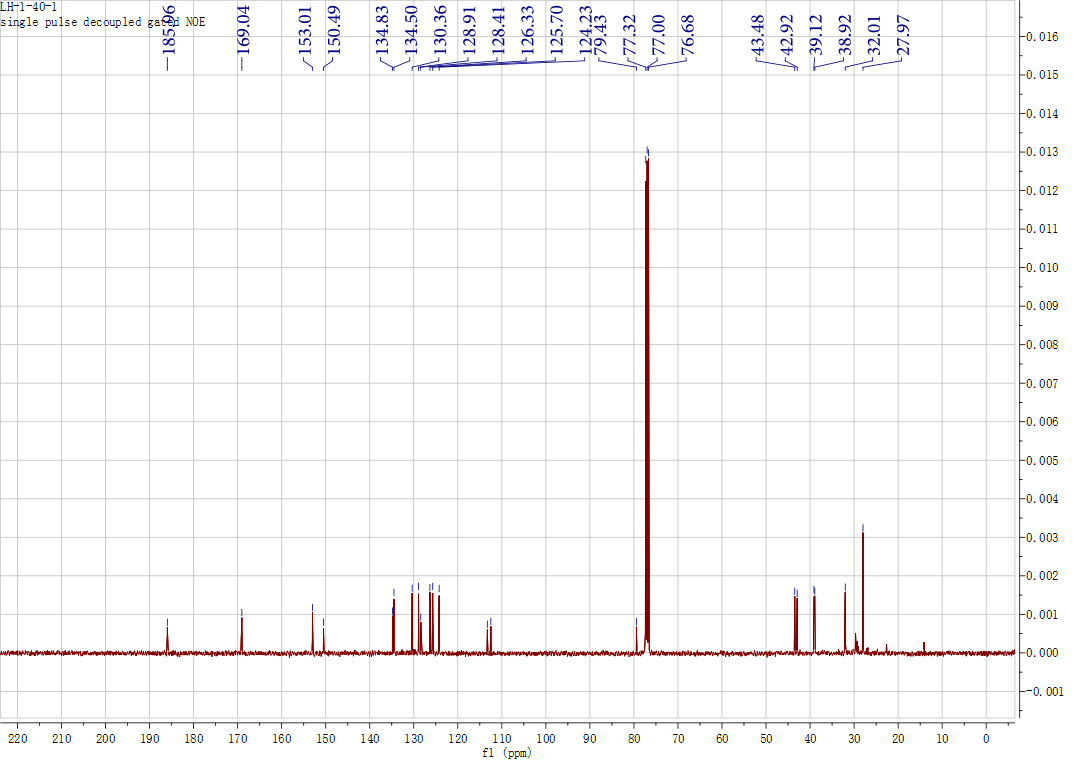


**Figure S3**. ^13^C NMR (400 MHz) spectra of DCI-H in CDCl_3_.


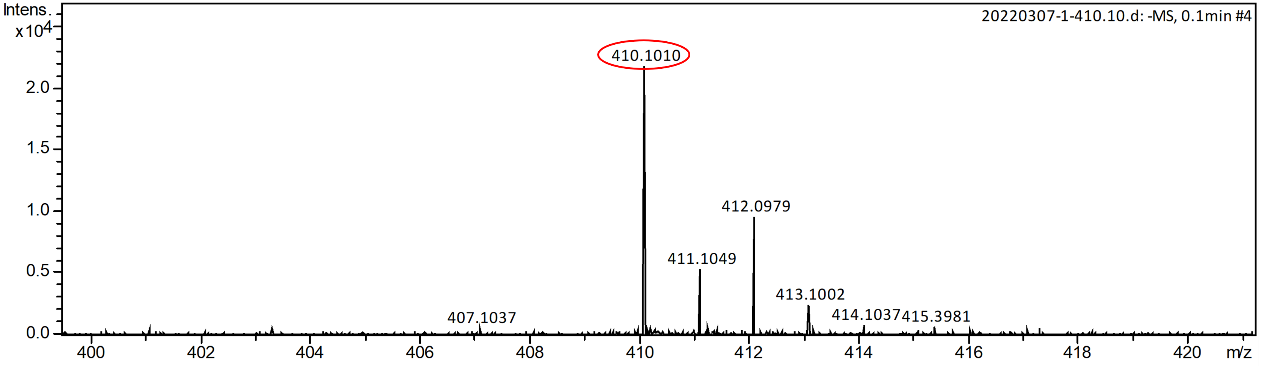


**Figure S4**. HRMS of DCI-H.


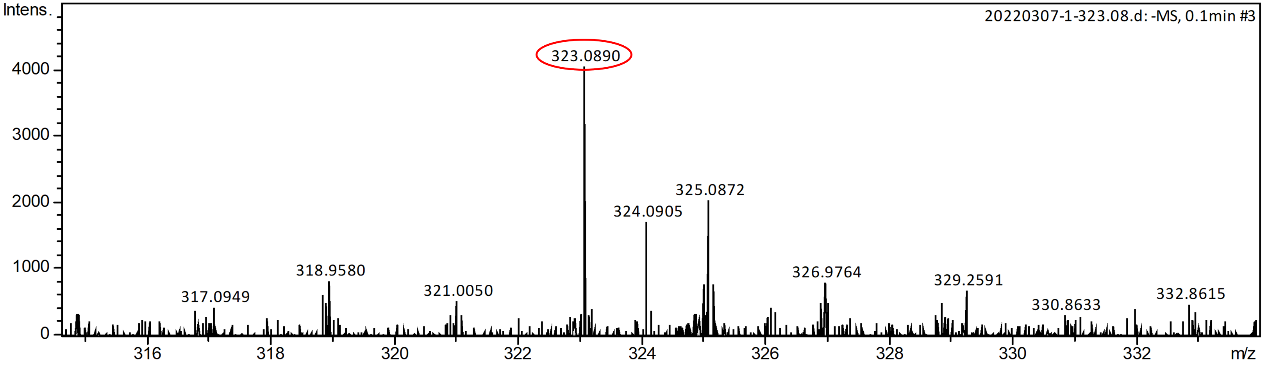


**Figure S5.** HRMS analysis of the reaction product of DCI-H and HOCl.

**5. The cytotoxicity of DCI-H in living cells**


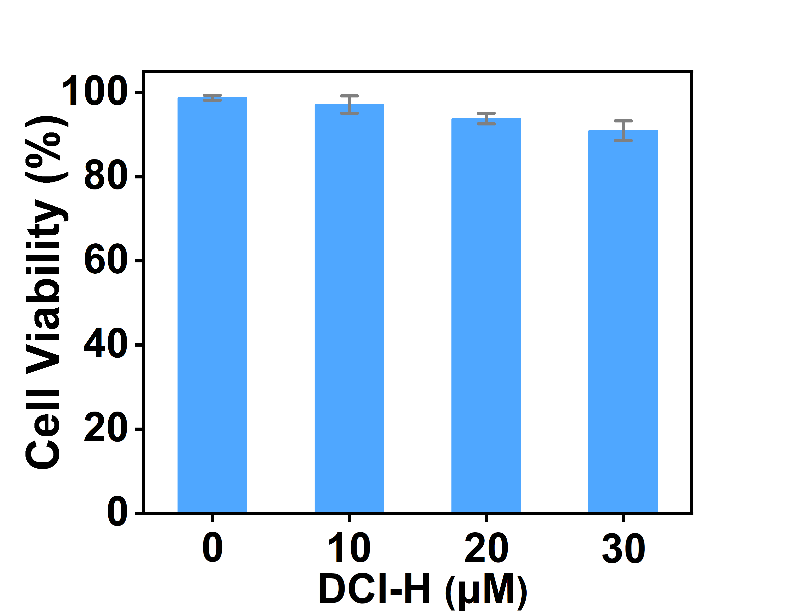


**Figure S6.** Cell viability of RAW264.7 cells in different concentrations of DCI-H was determined by CCK-8 assay. The experiments were repeated three times and the data were shown as mean (± S.D.).
